# ApoPred: Identification of Apolipoproteins and Their Subfamilies With Multifarious Features

**DOI:** 10.3389/fcell.2020.621144

**Published:** 2021-01-08

**Authors:** Ting Liu, Jia-Mao Chen, Dan Zhang, Qian Zhang, Bowen Peng, Lei Xu, Hua Tang

**Affiliations:** ^1^School of Basic Medical Sciences, Southwest Medical University, Luzhou, China; ^2^Center for Informational Biology, University of Electronic Science and Technology of China, Chengdu, China; ^3^Division of international Cooperation, Health Commission of Sichuan Province, Chengdu, China; ^4^School of Electronic and Communication Engineering, Shenzhen Polytechnic, Shenzhen, China; ^5^Central Nervous System Drug Key Laboratory of Sichuan Province, Luzhou, China

**Keywords:** apolipoprotein, identification, subfamily-classification, multiple features, machine learning

## Abstract

Apolipoprotein is a group of plasma proteins that are associated with a variety of diseases, such as hyperlipidemia, atherosclerosis, Alzheimer’s disease, and diabetes. In order to investigate the function of apolipoproteins and to develop effective targets for related diseases, it is necessary to accurately identify and classify apolipoproteins. Although it is possible to identify apolipoproteins accurately through biochemical experiments, they are expensive and time-consuming. This work aims to establish a high-efficiency and high-accuracy prediction model for recognition of apolipoproteins and their subfamilies. We firstly constructed a high-quality benchmark dataset including 270 apolipoproteins and 535 non-apolipoproteins. Based on the dataset, pseudo-amino acid composition (PseAAC) and composition of k-spaced amino acid pairs (CKSAAP) were used as input vectors. To improve the prediction accuracy and eliminate redundant information, analysis of variance (ANOVA) was used to rank the features. And the incremental feature selection was utilized to obtain the best feature subset. Support vector machine (SVM) was proposed to construct the classification model, which could produce the accuracy of 97.27%, sensitivity of 96.30%, and specificity of 97.76% for discriminating apolipoprotein from non-apolipoprotein in 10-fold cross-validation. In addition, the same process was repeated to generate a new model for predicting apolipoprotein subfamilies. The new model could achieve an overall accuracy of 95.93% in 10-fold cross-validation. According to our proposed model, a convenient webserver called ApoPred was established, which can be freely accessed at http://tang-biolab.com/server/ApoPred/service.html. We expect that this work will contribute to apolipoprotein function research and drug development in relevant diseases.

## Introduction

Apolipoprotein (Apo), a protein component of plasma lipoprotein, can bind and transport blood lipids to various tissues of the body for metabolism and utilization. It is mainly synthesized in the liver and partly in the small intestine (Yiu et al., 2020). A large number of studies have found that apolipoprotein gene mutation, the formation of different allelic polymorphisms, and further the generation of different phenotypes of apolipoprotein, can affect the metabolism and utilization of blood lipid, thereby triggering the occurrence and development of hyperlipidemia, atherosclerosis, cardiovascular and cerebrovascular diseases (Richardson et al., 2020). Millions of people around the world are suffering from apolipoprotein-related diseases (Cheng et al., 2019b; Fang et al., 2019).

Apolipoprotein includes A, B, C, D, E, L, F, H, M, N, and R subfamilies, each of which has different functions. Beyond the basic function of transporting lipids and stabilizing structure of lipoproteins, some types of apolipoprotein can activate lipoprotein metabolic enzymes and recognize receptors. Alterations in expression level, spatial structure, and function of apolipoproteins are closely related to a variety of diseases. For instance, the occurrence of hyperlipidemia and atherosclerosis is often accompanied by abnormal expression of high-density lipoprotein (HDL) and ApoA-I. Besides, the increased level of ApoB can raise the incidence of coronary heart disease. And ApoC-II can affect the uptake of triglyceride-rich lipoproteins by liver receptors, leading to the formation of human hypertriglyceridemia (Wolska et al., 2017). Moreover, ApoD is up-regulated in several human neurological disorders, such as Alzheimer’s disease (AD), Schizophrenia, Parkinson’s disease, and multiple sclerosis, and serves as an early diagnostic marker for a variety of cancers and neurological diseases (Martinez-Pinilla et al., 2015). Low level of ApoE in the brain and cerebrospinal fluid is associated with Alzheimer’s disease and other neurodegenerative diseases, as well as the early stage of many eye diseases (Mahley, 2016). In addition, ApoH participates in the coagulation process, and curbs ADP-mediated platelet aggregation by regulating adenylate cyclase activity; as a plasma inhibitory factor, it suppresses the activation of intrinsic coagulation pathway. Moreover, ApoM is a novel subtype of apolipoprotein discovered by Xu and Dahlback (1999). Studies suggested that ApoM takes a role in the antiatherogenic function of HDL through multiple pathways such as lipid metabolism, immune regulation, and anti-inflammatory effect (Arkensteijn et al., 2013). In patients with diabetes, the ApoM level is significantly reduced, and the rescue of ApoM level can decrease blood sugar level, increases insulin secretion, and improves insulin resistance, thereby serving as a predictor of the development of diabetes (Nojiri et al., 2014). Thus, correctly identify apolipoproteins and their subfamilies could provide important clues for understanding their function and roles in various of diseases.

Due to its biological function and association with multifarious diseases, apolipoprotein has gained increasingly more attention by researchers. Although more than 600 annotated apolipoproteins can be retrieved from the UniProt database, over 40,000 potential apolipoproteins are not annotated. However, identifying apolipoproteins in the vast amounts of data by biochemical assays will be a time-consuming and expensive task. Therefore, the research of apolipoprotein from the perspective of bioinformatics, with the help of a variety of statistical means and kinetic theory, can effectively narrow the target research scope.

In recent years, sequence alignment analysis has become the main bioinformatical study of apolipoprotein, which can reveal the evolution mode of apolipoprotein and predict the possible functional domains (Seda and Sedova, 2003; Weinberg et al., 2003; Toledo et al., 2004; Krisko and Etchebest, 2007; Deng et al., 2015). In 2000, Frank and Marcel (2000) analyzed the amino acid sequence composition and physicochemical properties of ApoA-I in 12 species. They found that the n-terminal of ApoA-I is highly conservative, while the c-terminal and the middle of the sequence display remarkable variation. Structural analysis suggested that the C-terminal is critical for lipid binding. Subsequently, Kiss et al. (2001) studied the functional similarity of ApoA-I in humans and chickens, declaring the correlation between the spatial structures of ApoA-I and lipid binding. Then, Gangabadage et al. (2008) studied the nuclear magnetic three-dimensional structure and kinetic properties of ApoC-III and simulated the binding structure of this protein and lipids. A recent study using sequence and structural alignment showed that the structure of ApoC-III is conserved in mammals. Bashtovyy et al. (2011) conducted sequence comparison of ApoA-I from 31 animals and found that there are conservative salt bridges in the first 30 residues and many conservative functional domains, revealing the relationship between apolipoprotein structure and function. Besides, Bandarian et al. (2016) studied the sequence variation of the ApoA-II gene and the correlation between this protein and serum level of HDL cholesterol. However, all above studies based on sequence or structure comparison have limitations. When facing a new sequence without homologs, these sequence alignment-based methods will be invalid. To solve the problem, in 2016, we designed a machine learning-based model to identify apolipoproteins (Tang et al., 2016) by using g-gap dipeptide feature extraction algorithm and LibSVM classifier. Nevertheless, this model has its own vulnerabilities, which cannot predict the subfamilies of apolipoproteins and the benchmark dataset built in the model is not large enough.

**FIGURE 1:**
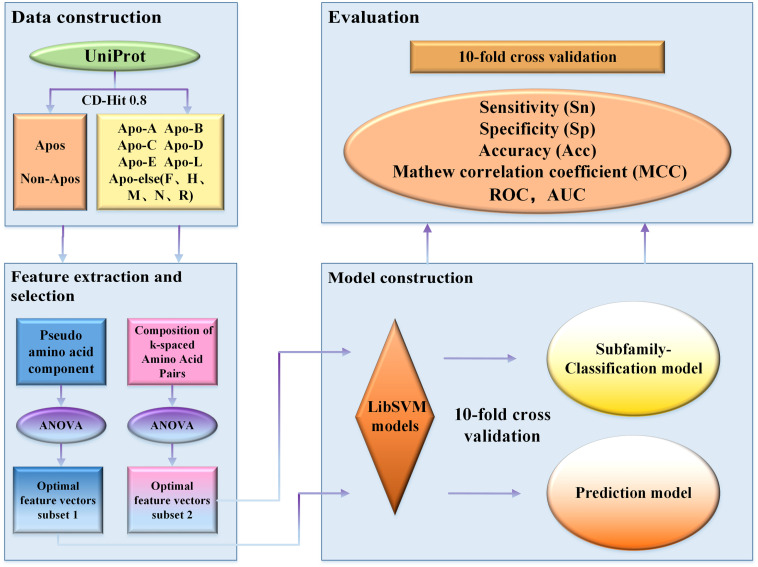
Flowchart of the proposed classification scheme.

To overcome the shortcomings mentioned above, we constructed a new benchmark dataset and developed a new model to distinguish apolipoproteins from non-apolipoproteins and further classified their subfamilies. Finally, based on the new model, we established a novel webserver called ApoPred, which can be freely accessible to all scholars. The whole process for the model construction was shown in Figure 1. This work can not only shed new light on the function of apolipoprotein, but also provide theoretical guidance for the further development of drug targets.

## Materials and Methods

### Benchmark Dataset

Establishment of a high-quality dataset is the key of constructing a prediction model (Liang et al., 2017; Zhang et al., 2017; Cui et al., 2018; Hasan et al., 2019a, b). All of our apolipoprotein sequence data was downloaded from the UniProt online database. To obtain the reliable dataset, all sequences are processed in the following steps:

a. Select the apolipoprotein sequences that have been annotated in the Swiss-Prot database.
b. Remove the sequences which contain undesirable characters: such as “B,” “J,” “O,” “U,” “X,” and “Z.”
c. Remove redundant sequences by setting the cutoff value of CD-HIT at 0.8

**FIGURE 2:**
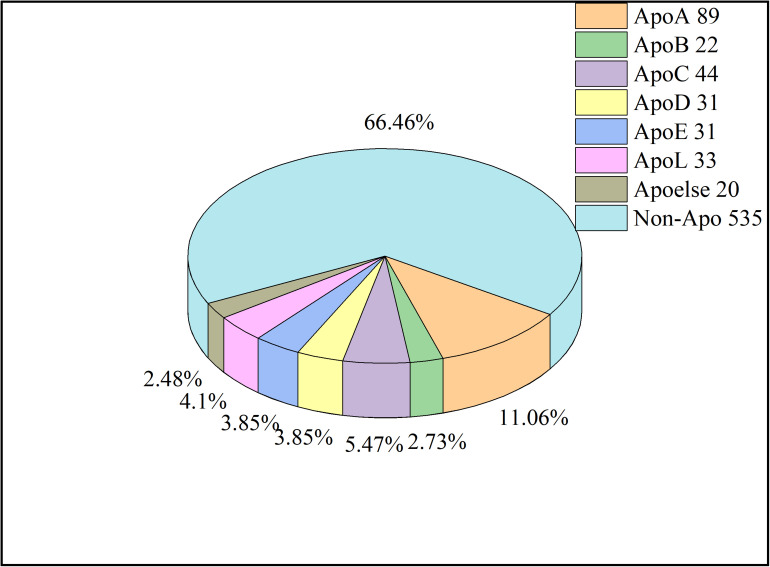
The pie chart of samples. The number after each Apo type indicates the sequence number. The Apoelse refers to the subfamilies including ApoF, ApoH, ApoM, ApoN, and ApoR.

For protein prediction, redundant sequences with similarity of higher than 40% are generally removed. Nevertheless, in this work, the cutoff value of CD-HIT was set at 0.8 in order to have enough sequences to train models. Thus, a total of 270 apolipoproteins remained. Due to the fact that the sample size of some subfamilies is too small to be compared statistically, we combined these subfamilies into a new class called Apoelse which contains 20 proteins. The details of apolipoprotein subfamilies were illustrated in Figure 2. Additionally, since apolipoproteins are mainly present in plasma, our negative samples (982 sequences) were selected from the non-apolipoproteins in plasma. To construct a reliable non-apolipoprotein dataset, we obtained 535 sequences with the sequence identity of less than 80%.

### Feature Expression

After constructing the dataset, we need to represent apolipoprotein sequence with a valid feature vector. It is obvious that the sequence, structure, and function are different between apolipoproteins and non-apolipoproteins, and among different apolipoprotein subfamilies. Generally, the differences are mainly manifested in long-term correlation, physicochemical properties, and amino acid composition. In this study, we tried a variety of feature extraction methods, and finally chose the optimal ones as the input vectors, namely pseudo-amino acid composition (PseAAC) and composition of k-spaced amino acid pairs (CKSAAP).

#### Pseudo-Amino Acid Composition (PseAAC)

PseAAC has been widely used in proteins prediction (Yang et al., 2016; Hasan et al., 2020b). It is defined by adding spatial structure and physicochemical properties to the amino acid frequency. The physicochemical properties considered in this work are hydrophobicity, hydrophilicity, mass, pK1 (alpha-COOH), pK2 (NH3), pI (at 25°C), rigidity, irreplaceability, and flexibility. Therefore, based on the formulation of Type II PseAAC, a protein sequence *P* with a total number of *L* amino acids can be described by a (20 + 9γ)-dimensional vector as follows:

(1) $$P={\left[A_{1},\cdots \,A_{20},A_{20+1},\cdots ,A_{20+9\,\gamma }\right]}^{T}$$

where “*T*” is a symbol of transpose operator. *A_i*(*i* = 1, 2, …, 20) represents the frequency of occurrence of 20 amino acids in protein *P*. *A_i*(*i* =  20 + 1, …, 20 + *n*γ) are the first to γth tire correlation factors of protein sequence which can be calculated according to the equations in references. *n* depends on the number of physical and chemical properties we used.

#### Composition of k-Spaced Amino Acid Pairs (CKSAAP)

The CKSAAP has also been used to analyze protein function (Ju and Wang, 2020). It calculates the frequencies of amino acid pairs separated by any *k* residues (*k* = 0, 1, 2, …, 5. The default maximum value of k is 5). Given a *k* value from 0 to 5, the number of occurrences of each *k*-spaced amino acid pairs can be determined from target sequences. Taking *k* = 0 as an example, we can get 20 × 20 residual pairs of 0-interval (i.e., AA, AC, AD, YY.). Thus, a given protein *P* can be formulated by a 400-Dimension vector as follows:

(2) $$P={\left(\frac{N_{A\,A}}{N_{t\,o\,t\,a\,l}},\frac{N_{A\,C}}{N_{t\,o\,t\,a\,l}},\frac{N_{A\,D}}{N_{t\,o\,t\,a\,l}},\cdots ,\frac{N_{Y\,Y}}{N_{t\,o\,t\,a\,l}}\right)}_{20\times 20}$$

where the *N*_*AA*_ represents the occurrence number of 0-interval residue pair AA in the protein sequence, and the *N*_*total*_ means the total number of 0-interval residual pairs in the given protein sequence. The value of each descriptor represents the frequency of the corresponding residue pair in the sequence. Then, when *k* = (1,2, …, 5), a protein *P* can be formulated as:

(3) $$P=(\frac{N_{A\,A\,0}}{N_{t\,o\,t\,a\,l\,0}},\cdots ,\frac{N_{Y\,Y\,0}}{N_{t\,o\,t\,a\,l\,0}},\frac{N_{A\,A\,1}}{N_{t\,o\,t\,a\,l\,1}},\cdots ,\frac{N_{Y\,Y\,1}}{N_{t\,o\,t\,a\,l\,1}},\cdots ,\;\frac{N_{A\,A\,k}}{N_{t\,o\,t\,a\,l\,k}},\cdots ,\frac{N_{Y\,Y\,k}}{N_{t\,o\,t\,a\,l\,k}}){}_{20\times 20\times \left(k+1\right)}$$

where the *N*_*AAk*_ denotes the occurrence number of *k*-interval residue pair AA in the protein sequence, and the *N*_*totalk*_ stands for the total number of *k*-interval residual pairs in the given sequence. For *k* = 0, 1, 2, 3, 4, and 5, the values of *N*_*totalk*_ are *P* – 1, *P* – 2, *P* – 3, *P* – 4, *P* – 5, and *P* – 6 for a protein of length *P*, respectively.

#### 188-Dimensional Feature Vectors

188D extracts sequence features based on 20 amino acid compositions and eight physicochemical properties (Ao et al., 2020). These features encode the primary sequence with 188-dimensional vectors (Li et al., 2019). Thus, the 188D of a given protein *P* is calculated as:

(4) $$P=\left(m_{1},\ldots ,m_{i},\ldots ,m_{20},C_{1},\ldots ,C_{i},T_{1},\ldots ,T_{i},D_{1},\ldots ,D_{i}\right)$$

The *m_i* is the frequency of 20 amino acids (in alphabetical order, ACDEFGHIKLMNPQRSTVWY) in the sequence. Then, the amino acids are classified into three groups according to each of the *n*(*n* = 1, 2, …, 8) physicochemical properties of proteins. For every single protein property, *C*_*i*_ is the frequency of occurrence of amino acids from the three groups respectively, yielding 3-dimension features; *T* describes the frequency of three types of dipeptides composed of two amino acids from different groups, which also generates 3-dimension features; *D* represents distribution of the three groups of amino acids at five specific points (first, 25%, 50%, 75%, and end in the sequence), through which the other 15-dimension features are extracted. In total, we obtain 20 + 8 × (3 + 3 + 15) = 188 dimensional features by this algorithm.

#### Dipeptide Composition (DPC)

The Dipeptide Composition is a commonly used algorithm for protein sequence description, giving 400 descriptors (Saravanan and Gautham, 2015; Manavalan et al., 2019a, b; Hasan et al., 2020a). It is defined as:

(5) $$D\,\left(r,s\right)=\frac{N_{r\,s}}{N-1}\;r,s\in \left(A,C,D,\cdots ,Y\right)$$

where *N*_*rs*_ is the number of dipeptides represented by amino acid types *r* and *s*, and the value of *N* stands for the length of a protein sequence.

### Feature Selection

In order to obtain the optimal feature subset and eliminate redundant and irrelevant features, analysis of variance (ANOVA) feature selection technology was adopted in this work.

ANOVA generally performs well in feature selection (Ding and Li, 2015; Kwon et al., 2020). Based on its definition, the features can be ranked by the corresponding *F*-value, as shown below:

(6) $$F\,\left(\theta \right)=\frac{S_{B}^{2}\,\left(\theta \right)}{S_{W}^{2}\,\left(\theta \right)}$$

where the *F*(θ) denotes the total variance, $S_{B}^{2}\,\left(\theta \right)$ and $S_{W}^{2}\,\left(\theta \right)$ are the variances between groups and within a group, separately.

The detailed formula are given in

(7) $$S_{B}^{2}\,\left(\theta \right)=\frac{1}{n_{i}-1}\,\sum_{j=1}^{n_{i}}{\left(x_{i\,j}-\frac{1}{n_{i}}\,\sum_{j=1}^{n_{i}}x_{i\,j}\right)}^{2}$$

(8) $$S_{W}^{2}\,\left(\theta \right)=\frac{1}{n-1}\,\sum_{i=1}^{k}\sum_{j=1}^{n_{i}}{\left(x_{i\,j}-\frac{1}{n}\,\sum_{i=1}^{k}\sum_{j=1}^{n_{i}}x_{i\,j}\right)}^{2}$$

where *x*_*ij*_ is the observations of the *j*th sample in the *i*th group, *k* is the number of group, *n_i* is the sample size of each group. And here *i* = 1, 2, …, *k.*

To determine the optimal feature combination, we employed incremental feature selection (IFS) (Zhu et al., 2019), which adds features to the feature subset in succession, and then study the influence of these features on the predicting performance of the constructed machine learning model. By strictly following the above steps, the optimal feature subset can be finally obtained when the maximum accuracy appeared.

### Model Construction by Support Vector Machine (SVM)

SVM is a supervised learning method which has been widely applied in statistical classification and regression analysis (Manavalan and Lee, 2017; Xu et al., 2018a, b; Basith et al., 2019; Lai et al., 2019; Manavalan et al., 2019c; Wang et al., 2019; Yang W. et al., 2019; Dao et al., 2020b). Proposed in 1964, SVM developed rapidly after 1990s and derived a series of improved and extended algorithms which have been performed in pattern recognition such as portrait recognition and text classification (Qin and He, 2005). SVM uses hinge loss function to calculate empirical risk and adds regularization terms in the solution system to optimize structural risk. Besides, SVM can build a hyperplane to carry out non-linear classification through kernel function (Bredesen and Rehmsmeier, 2019). Due to its good performance in non-linear classification, we employed SVM in this study. We adopted a tool of SVM, the LibSVM package, which can be obtained from: https://www.csie.ntu.edu.tw/~cjlin/libsvm. Grid search was used to optimize the parameters C and γ.

### Performance Evaluation

Cross-validation is an objective method for evaluating the performance of predictors (Cheng and Hu, 2018; Cheng et al., 2019a; Tahir and Idris, 2020). In our study, 10-fold cross-validation was applied to assess our prediction model. Sensitivity (Sn), specificity (Sp), accuracy (Acc), and Mathew correlation coefficient (MCC) are commonly used to measure the performance of classifiers (Xu et al., 2018c, 2019; Boopathi et al., 2019; Huang et al., 2019; Liang et al., 2019; Stephenson et al., 2019; Yang H. et al., 2019; Basith et al., 2020; Dao et al., 2020a; Hasan et al., 2020c; Zhao et al., 2020), and can be defined as follows:

(9) $$S\,n=\frac{T\,P}{T\,P+F\,N}$$

(10) $$S\,p=\frac{T\,N}{T\,N+F\,P}$$

(11) $$A\,c\,c=\frac{T\,P+T\,N}{T\,P+T\,N+F\,P+F\,N}$$

(12) $$M\,C\,C=\frac{\left(T\,P\times T\,N\right)-\left(F\,P\times F\,N\right)}{\sqrt{\left(T\,P+F\,P\right)\,\left(T\,P+F\,N\right)\,\left(T\,N+F\,P\right)\,\left(T\,N+F\,N\right)}}$$

where *TP* and *TN* are the correctly predicted positive and negative samples, respectively; *FP* and *FN* are the falsely predicted positive and negative samples, respectively.

The Receiver Operating Characteristic (ROC) curve can intuitively represent the influence of any threshold on the generalization of the constructed prediction model (Wang et al., 2020). Generally, the closer the ROC curve is to the point of (0, 1), the higher the recall of the model is. Furthermore, the area under ROC curve (AUC) is the important numerical indicator of ROC curve, which ranges from 0 to 1. The performance of classifier is positively related to the value of AUC. Thus, we also used ROC curve and AUC to evaluate the model.

## Results

### The Accuracy for Apolipoproteins Prediction

We trained SVM with the different feature extraction strategies. And the best feature extraction method was selected to construct the final prediction model. In each feature extraction, feature selection was applied to achieve the optimal feature subset. In our research, a total of four different feature extraction strategies were examined. Results are recorded in Table 1.

**TABLE 1:** The results of four feature extraction methods in prediction of apolipoprotein.

**Feature**	**Acc (%)**	**Sn (%)**	**Sp (%)**	**MCC**	**Number**
CKSAAP (k = 4)*^*a*^*	97.02	96.67	97.20	0.93	180
DPC*^*b*^*	96.40	**97.41**	95.89	0.92	381
PseAAC*^*c*^*	**97.27**	96.30	**97.76**	**0.94**	**70**
188D*^*d*^*	95.53	92.59	97.01	0.90	182

**^*a*^Represents composition of k-spaced amino acid pairs. ^*b*^Means the dipeptide composition. ^*c*^Is Pseudo-amino acid composition. ^*d*^Stands for 188-dimensional feature vectors that is cited from literature (Liao et al., 2016).* The bold values indicate the best performances of the methods.*

As shown in Table 1, the PseAAC achieved the highest Acc of 97.27% among four feature extraction methods. In addition, it also gained the best *MCC* of 0.94 and *Sp* of 97.76%. This suggests that apolipoprotein and non-apolipoprotein can be predicted satisfactorily according to the differences in their sequences.

**FIGURE 3:**
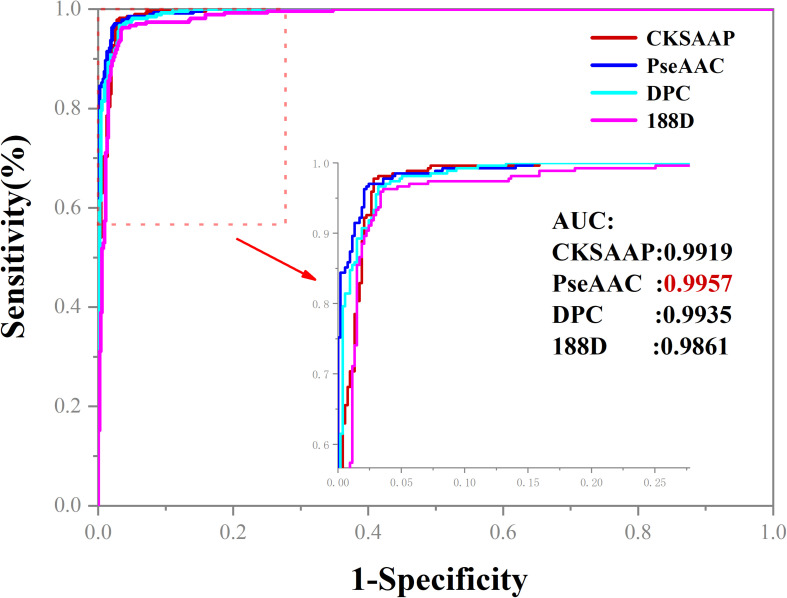
The ROC curves of four feature extraction methods. When PseAAC was applied to construct the model for apolipoprotein prediction, the AUC reaches the peak of 0.9957.

To further evaluate the predictive performance of our models, we plotted the ROC curves in Figure 3. Obviously, PseAAC is the best one among the four features for apolipoprotein prediction because it could produce the AUC of 0.9957.

### The Accuracy for Apolipoprotein Subfamily Classification

Up to now, this is the first machine learning work for apolipoprotein subfamily classification. By identifying the subfamilies of apolipoprotein, we aimed to provide more comprehensive understanding of apolipoproteins’ function. We investigated the performances of three kinds of features: CKSAAP, DPC, and PseAAC with feature selection. Results are listed in Table 2.

**TABLE 2:** The results of four feature extraction methods in subfamily classification of apolipoprotein.

**Feature**	**CKSAAP**		**DPC**	**PseAAC (γ = 10)**	**188D**
	**K = 3**	**K = 4**			
ACC (%)	**95.93**	**96.67**	94.44	91.11	90.37
Number*^*e*^*	169	763	142	56	53

**^*e*^Represents the optimal number of features left after feature selection.* The bold values indicate the top two performances of the CKSAAP.*

**FIGURE 4:**
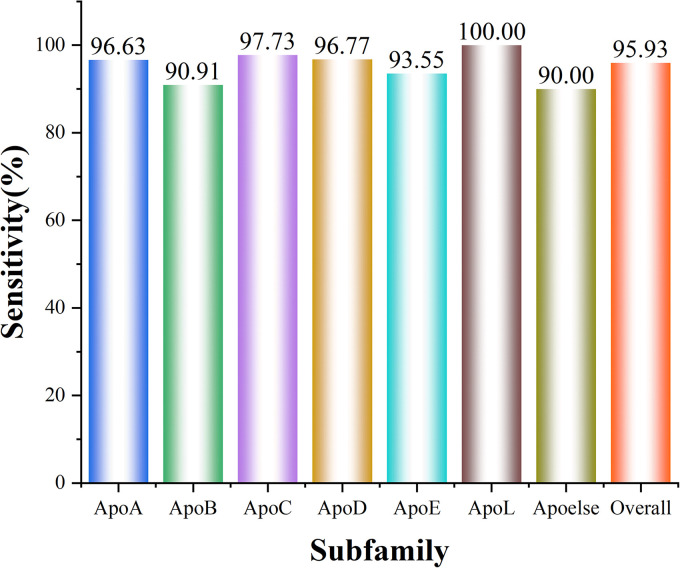
A histogram showing the classification sensitivity of each subfamily. ApoL has the highest Sn of 100%, while Apoelse gets the relatively lowest Sn of 90.00%. And the total Sn is 95.93%.

As presented in Table 2, the best accuracy of 96.67% was obtained via CKSAAP with the *k* = 4. However, such high accuracy was produced at the cost of a high-dimension feature vector (763 D). From the table, one may notice that when *k* of CKSAAP was set to 3, the overall accuracy is 95.93% which is slightly lower than that of *k* = 4. Whereas, the dimension of input feature decreases dramatically from 763 to 169. Thus, the model constructed on CKSAAP (*k* = 3) is more robust and reliable. The prediction accuracy for each subfamily in 10-fold cross-validation is illustrated in Figure 4.

Different subfamilies of apolipoprotein have different functions and play distinct roles in the metabolism and physiological process of lipoprotein. Therefore, the subfamily classification of apolipoprotein is particularly significant. In our model, the highest accuracy of 100% was obtained for the ApoL subfamily. However, for Apoelse prediction, the accuracy is 90.00% which is the lowest among all subfamilies. The reason for this low accuracy is that Apoelse contains several types of subfamilies and the apolipoproteins in these subfamilies are not very similar in feature space. Such phenomenon also demonstrates that the apolipoproteins in different subfamilies possess different intrinsic sequence characteristics, structure, and function. Given this, our subfamily classification model of apolipoprotein is stable and reliable.

### Webserver

For the sake of most scholars, we established a user-friendly webserver called ApoPred. Users can browse the server homepage at http://tang-biolab.com/server/ApoPred/service.html. And the webserver is guaranteed to work properly for at least 2 years. A detailed guide on how to use the webserver is given below.

**FIGURE 5:**
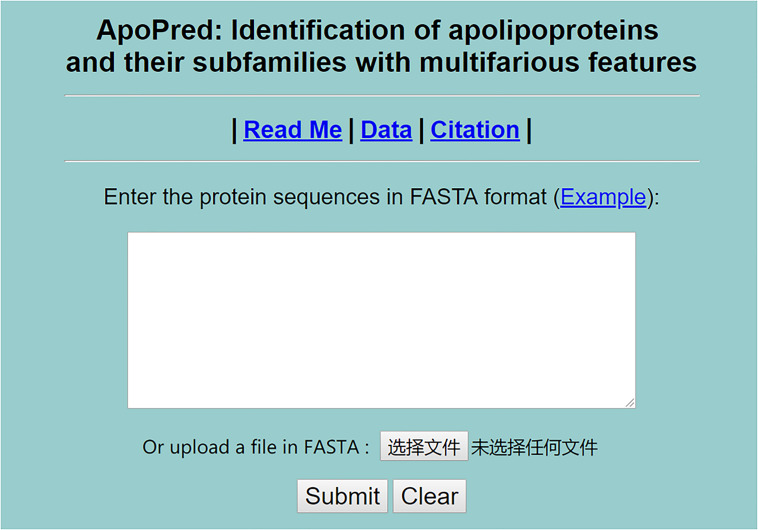
The top page of the ApoPred webserver at http://tang-biolab.com/server/ApoPred/service.html.

On the home page of ApoPred, as shown in Figure 5, the Read Me button provides a brief introduction of the predictor and warnings when using it. Click the Data button, and the benchmark dataset that we built can be freely downloaded.

The users can input or paste the query amino acid sequences into the input box in FASTA format. The Example button supplies users with our example sequences in FASTA format. When clicking the Submit button, users can view the results of apolipoproteins identification and their subfamily classification.

## Discussion

It is acknowledged that apolipoprotein has crucial effect on regulating lipoprotein metabolism, and variations in the expression level. Spatial structure and function of apolipoprotein are associated with numerous diseases. Nevertheless, lack of intensive bioinformatical analysis on the function and classification of apolipoprotein restricted its application on drug targets for the associated diseases.

**FIGURE 6:**
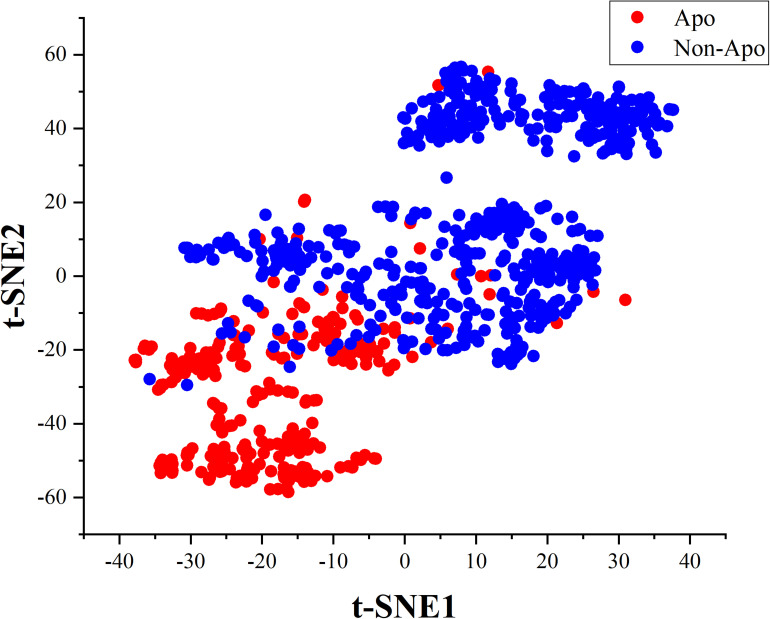
Cluster analysis of apolipoproteins and non-apolipoproteins.

**FIGURE 7:**
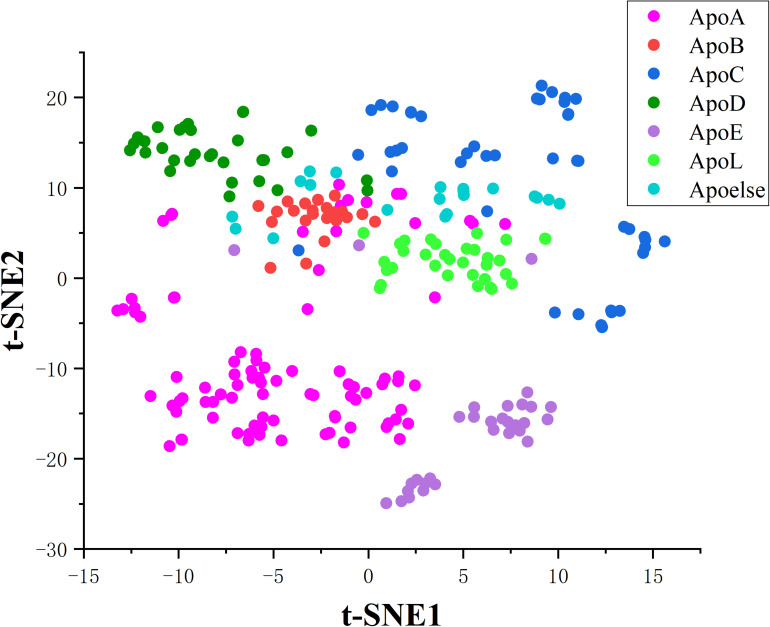
Cluster analysis of apolipoprotein subfamilies.

In this work, we innovatively applied the correlation features obtained from residues sequence on constructing a two-tier classifier to identify apolipoproteins and their subfamilies. All the corresponding results and models stem from a reliable benchmark dataset which have been verified by biochemical experiments. Besides, the correlation of amino acids residues contains key genetic information. We consequently compared four feature extraction strategies describing the association between apolipoprotein amino acids and selected the optimal features by incorporating ANOVA into IFS. The PseAAC employed in apolipoprotein prediction model has been widely used in various fields of computational proteomics (Feng et al., 2013; Yang et al., 2016; Long et al., 2017). Another feature expression of CKSAAP used for subfamily classification is also a convenient tool in bioinformatics (Wang et al., 2016; Li et al., 2017; Cheng, 2019). The prediction models based on these features achieved encouraging results in 10-fold cross validation, which are demonstrated by cluster analysis via t-distributed stochastic neighbor embedding (t-SNE). The visualization results are shown in the Figures 6, 7.

After feature extraction and selection procedure, 70-dimensional feature vectors were generated, which were reduced to 2-dimensional by t-SNE algorithm to facilitate clustering analysis. t-SNE is a common technique for dimensionality-reduction and visualization of high-dimensional data. As displayed in Figure 6, apolipoproteins are well separated from non-apolipoproteins. This illustrates that the features of PseAAC have promising performance in apolipoprotein classification. Similarly, as shown in Figure 7, the first six subfamilies, namely, ApoA, ApoB, ApoC, ApoD, ApoE, and ApoL, are obviously separated, while the seventh class, Apoelse, partly overlaps with ApoB, ApoC, ApoD, and ApoL, possibly because the seventh class is not a pure subfamily but a combination of ApoF, ApoH, ApoM, ApoN, and ApoR.

In addition, due to the size of the dataset provided by UniProt, our model does not conduct independent data validation. However, the validation of independent data will be carried out in our future work by collecting more apolipoprotein data.

In a word, based on feature extraction and selection algorithm, our models performed excellently in apolipoprotein recognition and subfamily-classification.

## Conclusion

In this research, a practical tool, named ApoPred, was established to identify potential apolipoproteins and their subfamilies, providing a new theoretical basis for apolipoprotein function research and a new approach for drug target development. We have constructed the latest, high-quality, and reliable dataset to date, which is potentially to be conducted as the standard dataset for apolipoprotein research. We also successfully applied strategies of feature extraction and selection to obtain high-accuracy and robust classification models, which will facilitate further research of apolipoprotein function and drug targets for the relevant diseases. In the future, we will construct a more robust and precise model based on deep learning (Cao et al., 2017; Sunil et al., 2017; Si et al., 2020; Tomasz et al., 2020) and fusion features to identify apolipoproteins.

## Data Availability Statement

The original contributions presented in the study are included in the article/supplementary materials, further inquiries can be directed to the corresponding author/s.

## Author Contributions

HT conceived and designed the study. TL conducted the experiments. TL, J-MC, DZ, QZ, and BP implemented the algorithms. DZ established the web server. TL, LX, and HT performed the analysis and wrote the manuscript. All authors read and approved the final manuscript.

## Conflict of Interest

The authors declare that the research was conducted in the absence of any commercial or financial relationships that could be construed as a potential conflict of interest.

## Abbreviations

188D: 188-dimensional feature vectors
ANOVA: analysis of variance
Apo: apolipoprotein
CKSAAP: composition of k-spaced amino acid pairs
DPC: Dipeptide Composition
IFS: incremental feature selection
PseAAC: pseudo-amino acid composition
SVM: support vector machine.
